# Co-curricular engagement among engineering undergrads: do they have the time and motivation?

**DOI:** 10.1186/s40594-023-00410-1

**Published:** 2023-04-05

**Authors:** Andrew Olewnik, Yunjeong Chang, Mengchen Su

**Affiliations:** 1 Department of Engineering Education, University at Buffalo, 140 Capen Hall, Buffalo, NY 14260 USA; 2 Department of Learning and Instruction, University at Buffalo, 578 Baldy Hall, Buffalo, NY 14260 USA; 3 Center for Applied Research and Educational Improvement, University of Minnesota, 460A Learning and Environmental Sciences Building, 1954 Buford Ave, St. Paul, MN 55108 USA

**Keywords:** Co-curricular, Engineering, Motivation, Expectancy value theory

## Abstract

**Background:**

Co-curricular activities are often touted as valuable STEM learning opportunities in higher education settings. Particularly in engineering, industry encourage and seek students with co-curricular experiences. However, many engineering undergraduates do not regularly participate in those experiences. Some researchers have suggested that the rigors of the curriculum leave little time for co-curriculars. Yet, little research has empirically examined the reality of the undergraduate students’ involvement in co-curriculars. Thus, as an initial study, we situated our study in a large public university to explore students’ motivations for co-curriculars. In this paper we report on our efforts to understand student perceptions about the value and costs of that involvement. We considered how undergraduate engineering students used their time and what motivated them to engage (or not) in co-curriculars using Expectancy-Value Theory (EVT). Students’ motivation was investigated with a quantitative research methodology and complemented by interview data.

**Results:**

Results of our motivation survey show that students who participated in co-curriculars perceived less cost than those who never participated. We also found that the achievement values of co-curriculars does not necessarily motivate student involvement. Interview data were used to further interpret quantitative data results.

**Conclusions:**

In the context of study findings and existent literature, we discuss several implications for future research and practice. First, we argue for a more granular investigation of student time use and its impact on co-curricular participation. Second, despite the potential for high impact outcomes, students who have never participated perceived high cost for co-curricular engagement. Those perceptions may aggravate inequitable engagement of student populations, including historically marginalized populations in the STEM field. Third, students do not necessarily associate co-curricular experiences with the types of achievement values and learning that institutions, alumni, and industry might consider most important. Thus, to build and support co-curricular programs that provide the holistic educational experiences and learning that are anticipated, research that supports design of co-curricular programs and policies to improve engagement and persistence in those programs for all students is necessary.

**Supplementary Information:**

The online version contains supplementary material available at 10.1186/s40594-023-00410-1.

## Introduction

Engineering education has had continued calls for reform over the past two decades to better prepare students for engineering practice in the twenty-first century (National Academy of Engineering, 2005). Calls for creative design thinkers, with the ability to engage the technical and non-technical aspects of interdisciplinary problems are examples of reforms that have been recognized for some time (Dym et al., 2005; Jonassen et al., 2006; National Academy of Engineering, 2005). As a core of reform, industry leaders suggested that undergraduate engineers build competency and a portfolio of work by engaging in authentic multidisciplinary and collaborative experiences (National Academy of Engineering, 2013), like those found in many co-curricular environments. Thus, we see co-curricular engagement as an important opportunity for undergraduate students.

Co-curriculars are “structured learning activities that complement the formal curriculum (and more often than not do not count for credit or toward graduation)” (Rutter & Mintz, 2016). They can include a wide array of activities that occur outside of the curriculum, and the interest in this research focuses on co-curricular activities that are like the profession. This includes experiences and activities that complement coursework in the major without being directly tied to a specific course (Simmons et al., 2017). For example, participation in a student engineering club focused on professional development activities or a technical competition provides learning opportunities that are more directly applicable to the profession than would a co-curricular like an intramural sport. Co-curriculars in the form of makerspaces, undergraduate research, and student engineering clubs are forms of co-curriculars that inform this work, as they occur on campus, with input and support from institutions.

One theme that emerges from the NAE panel report (National Academy of Engineering, 2013) is that engineering curricula and institutions are generally not agile enough to broadly enact the types of reforms suggested by the panel. We contend that this lack of agility has been an issue for some time, and it is one reason that co-curricular engagements, such as student clubs, internships, and co-ops, have played such an important role in the professional preparation of engineering students. For example, on the campus where this research was conducted, select students work as part of a co-curricular lab on the design of nanosatellites with funding and mentoring from the Air Force. In such an authentic environment, project requirements, engineering activities, and the nature of interactions is better aligned with the profession (Trevelyan, 2007). This may be because, compared with a classroom, the forms of accountable disciplinary knowledge (Stevens et al., 2008) found in the nanosat lab are better aligned with those expected in the profession. Thus, co-curriculars are recognized among the “high-impact” experiences in higher education (Kuh, 2008) that enable students to supplement technical-focused classroom learning with experiences that integrate professional competencies necessary for practice (Gilbuena et al., 2015; Miller et al., 2018; Passow & Passow, 2017). They foster development of technical and professional competencies in ways that can be difficult, if not impossible, to replicate authentically in a classroom.

Most institutions of higher education, reinforced by alumni and industry sentiment, encourage students to pursue co-curricular opportunities as part of a holistic educational experience. Such co-curricular activities enable students to supplement their learning through experiences that make them more competitive candidates for employment (Miller et al., 2018). There are a variety of positive developmental outcomes of co-curriculars described in the literature and evidence that involvement in co-curriculars improve experiences and retention of historically marginalized groups (Carter et al., 2016; Hinkle & Koretsky, 2019; Huang & Chang, 2004; National Academies of Sciences, 2017). Still, participation in the field is not consistent with representation in the general population (Roy, 2019). Consequently, encouraging and supporting co-curriculars may be particularly beneficial in institutional efforts to recruit and retain students from marginalized groups and broadening participation in the profession. However, the actual learning and competency development that occurs through co-curriculars, and how it might vary across student populations and disciplines is not well-understood. Furthermore, the voluntary nature of co-curriculars and the rigorous nature of the engineering curriculum can be overwhelming for students leading to a lack of time for co-curricular engagement (Lichtenstein et al., 2010; Simmons et al., 2017). This makes it difficult to convince students to participate and to inform institutions in developing support models.

### Study motivation: overview of preliminary study on student available time

**Fig. 1:**
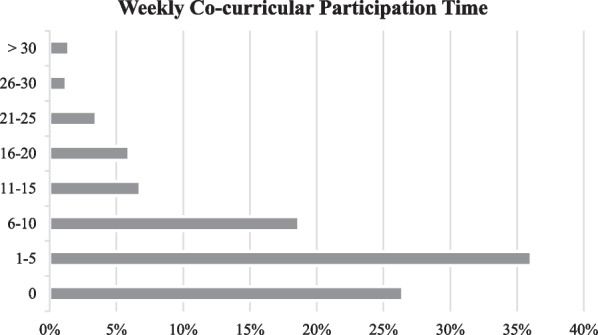
Time spent on co-curriculars as reported by students in the 2017 NSSE (*N* = 488)

In the context of the challenges for engineering undergraduate participation reported in the literature, we sought to better understand these challenges within our own institution. These challenges are exemplified in lower-than-expected engagement and lack of persistence in a makerspace setting, and consistent reports from engineering student club leaders that it is difficult to attract and retain members. These anecdotal reports and observations are consistent with 2017 National Survey of Student Engagement (NSSE) data for our institution. As shown in Fig. 1, most engineering undergraduates at our institution reported low or no engagement in co-curriculars, with 62% of respondents reporting 5 h or fewer of co-curricular involvement. (Note: NSSE data make no distinction between co-curriculars and extracurriculars as we have defined them previously).

Given the dearth of time studies in the literature, in recent work we sought to understand what available time engineering students at our institution (a large public, research intensive university in the Northeastern United States with an undergraduate engineering population of ~ 4500 students) might have for co-curriculars. Using the semester schedules for engineering undergraduate students and National Survey of Student Engagement (NSSE Overview, 2021) supplemented by data from the Bureau of Labor and Statistics American Time Use Study (2021), we estimated that the “typical” engineering undergraduate at our institution has on the order of 12–25 total hours of residual time during the weekdays with at least one 2-h long opening during the week (Olewnik & Sreeram, 2021). Based on the apparent difference in potential time for co-curricular activities and the actual time spent on co-curriculars as reported by students (Fig. 1), several questions arise. Some are related to developing a more individualized understanding of time constraints, while others are related to student motivation and their perceptions of the cost–benefit tradeoff for co-curriculars.

The study reported here is focused on this latter issue, in which the fundamental research question is: *Which motivational factors might explain students’ engagement in co-curricular activities?* Through a pilot survey based on Expectancy-Value Theory (Eccles & Wigfield, 2002) we explored student perceptions of the benefits and costs of co-curricular involvement.

If co-curricular experiences indeed foster a holistic educational experience that can better prepare students for the profession, it is important to encourage and increase participation. The study reported here fits within a broader research context that aims to (1) increase understanding of the factors that affect engineering student engagement and learning in co-curriculars, and (2) operationalize that increased understanding to inform engineering student decisions related to educational choice, and programmatic activities, spaces, and relevant support structures to improve student engagement and persistence in co-curriculars.

## Literature review

In this section, we consider the research literature related to the potential educational value and challenges of co-curriculars. We specifically focus on co-curricular activities with an integrated technical element. A cross-disciplinary study of undergraduate student involvement in academic and co-curricular activities found positive correlation between academic and co-curricular involvement on cognitive and affective growth (Huang & Chang, 2004). Furthermore, the study authors concluded that participation in co-curriculars need not come at the expense of traditional, academic engagement. Instead, other intrapersonal and contextual factors should be considered (e.g., time management) if participation in co-curricular involvement is perceived as negatively impacting curricular engagements (Huang & Chang, 2004).

We focused on co-curricular activities with an integrated technical element that can be engaged on campus during the academic year—makerspaces, undergraduate research, and student engineering clubs. Makerspaces are “collaborative work space inside a school, library or separate public/private facility for making, learning, exploring and sharing that uses high tech to no tech tools” (*What Is a Makerspace?*, 2015). In engineering academic settings, they often integrate elements of community makerspaces (i.e., anyone can access resources to support their creative development) with elements of traditional engineering programs (e.g., machine shops, computer labs) (Torralba & Rouse, 2019). Undergraduate research (UR) experiences are a well-researched topic and include both curriculum-integrated and co-curricular models. Engineering student clubs are often associated with professional organizations—e.g., Society of Automotive Engineers (SAE), American Society of Civil Engineers (ASCE)—that promote professional development through technical project competitions, conferences, and networking events.

### Value of technical focused co-curricular engagement

A range of benefits associated with co-curricular activities have been described in the literature and should be central to encouraging student participation. These include improved retention and persistence in the discipline (National Academies of Sciences, 2017), gains in cognitive skills (Carter et al., 2016; Hinkle & Koretsky, 2019), improvements in professional competencies (Carter et al., 2016; Shehata, 2015; Young et al., 2014), and positive impacts on affective aspects, such as motivation and self-efficacy (Hilton et al., 2018; Torralba & Rouse, 2019).

As it relates to cognitive skills, involvement in makerspaces provides opportunities for innovative problem solving (Andrews et al., 2021). These opportunities are inherent to their capacity for content agility, learning flexibility, and non-traditional access to STEM learning (Halverson & Sheridan, 2014). With more authentic contexts, undergraduate research (UR) can provide a deeper understanding of scientific findings (Zydney et al., 2002), and student clubs can provide opportunities for creativity and experimentation (Hinkle & Koretsky, 2019).

With respect to professional competencies, Carter et al. (2016) found that involvement in UR is a significant predictor of communication skills; students with such experiences reported higher levels of communication skills compared to their non-UR peers. A study of engineering alumni found that those who participated in a structured UR program reported greater enhancement of speaking skills and career goals when compared to their non-UR peers (Zydney et al., 2002). Student clubs can lead to deep technical experience and industry-aligned practices, as well as effective communication and consideration of social and cultural context (Hinkle & Koretsky, 2019). Other studies focused on the co-curricular experiences of African American students reported gains in professional skills, such as teamwork, reflective behavior, and communication (Garrett et al., 2021; Young et al., 2014).

For affective development, co-curricular involvement can improve sense of belonging and confidence in abilities. According to a consensus study from the National Academies of Sciences, Engineering, and Medicine, there is robust evidence demonstrating that involvement in undergraduate research improves retention within STEM fields, including individuals from historically marginalized groups (National Academies of Sciences, 2017). Rodriguez Amaya et al. (2018) investigated the undergraduate research experience of marginalized groups and found that it supports student retention and success. Research on makerspaces has found that engagement in those spaces can lead to positive impacts on self-efficacy and retention among students from marginalized populations (Andrews et al., 2021; Hilton et al., 2018; Tomko et al., 2021; Torralba & Rouse, 2019; Vongkulluksn et al., 2018). However, it is critical that they support broad views on what counts as making and attend to other important elements that may undermine involvement of marginalized groups (Andrews et al., 2021; Tomko et al., 2021; Vossoughi et al., 2016). Positive changes are possible even if the level of participation remains low across the semester (Hilton et al., 2018). Positive gains in the affective dimension are critical to supporting persistence in STEM, where students often lose interest when relevance or personal interest is not developed (Guo et al., 2015; Harackiewicz et al., 2014). Students from marginalized groups—who have higher dropout rates than their non-marginalized peers (Roy, 2019)—have been found to benefit more from high impact experiences, than their majority peers in terms of curricular performance and persistence (Kuh, 2008; National Academies of Sciences, 2017). Thus, encouraging and supporting co-curriculars may be particularly beneficial in institutional efforts to recruit and retain students from historically underrepresented groups in STEM.

Co-curriculars appear to have untapped potential to support student learning in ways that are well-aligned with industry expectations. However, despite apparent value, it appears that many students are disinclined to participate.

### Challenges to co-curricular engagement

The literature suggests a few critical challenges to understanding and assessing the value and place of co-curriculars within the undergraduate experience. These challenges negatively impact our ability to effectively design and support co-curriculars and may undermine students’ motivation to participate.

#### Learning outcomes by co-curricular type

First, the educational benefits reported in the literature are mixed and not generalizable across co-curricular activities or student populations. While some beneficial learning outcomes may be expected, others might not. For instance, while involvement in undergraduate research has been found to have positive impacts on technical communication, other professional competencies such as teamwork and leadership are not significantly affected (Carter et al., 2016). In other cases, while some studies show that more co-curricular engagement leads to greater gains, there may be diminishing returns or even unintended downsides from over-involvement. For example, co-curricular engagement can have a positive impact on ethical decision-making and leadership, and can reinforce classroom learning (Burt et al., 2011; Wilson et al., 2014). However, over-involvement (i.e., spending too much time on a co-curricular) can create academic pressures that lead to unethical behavior (Burt et al., 2011).

The National Academies note that there remains a need for more systematic research into the outcomes of undergraduate research experiences to improve undergraduate training (National Academies of Sciences, 2017). Hinkle and Koretsky (2019) suggest that the ways in which student clubs help in professional formation and the specific forms of learning are poorly understood. In addition, considerations of the infrastructure, support structures, and their scalability are all factors that require additional study across co-curricular types (Hinkle & Koretsky, 2019; Ludwig et al., 2017; Torralba & Rouse, 2019). The types of co-curricular and extracurricular engagement selected by students and their level of engagement therein has been found to differ by gender and ethnicity and is not necessarily guided by the benefits found in the literature (Simmons et al., 2018). There are numerous factors that impact the benefits of co-curriculars that can vary greatly from one institution to another and require intentional support structures to maximize benefits (Lee & Matusovich, 2016). This might explain why co-curricular benefits for one group at one institution are not necessarily observed at another institution.

#### Student time constraints

A second challenge is that the benefits that might be obtained from co-curricular activities are constrained by the time available for students to participate. The demands of the engineering curriculum make it difficult for many students to pursue co-curriculars (Lichtenstein et al., 2010; Miller et al., 2018; Simmons et al., 2017, 2018). Lichtenstein et al. (2010) analyzed NSSE data from the early 2000s for multiple institutions. They noted that first-year and senior engineering students—the populations surveyed by NSSE—reported spending their time similar to students in other majors except for significantly more time spent preparing for class and less time working for pay off campus. The authors concluded that “the engineering curriculum creates demands that force students to make choices between acquiring practical (and highly marketable) skills during college in exchange for missing out on various educationally enriching experiences.”

Based on this fundamental conclusion, Simmons et al. (2018) conducted a preliminary study to understand engineering students’ participation in “out-of-class” (co-curricular and extracurricular) activities from the students’ perspectives. The study found that the top activities reported by engineering students include sports, job (with no distinction between engineering and non-engineering work), and design competition teams. The authors concluded that it is concerning to see “a comparative lack of engagement in co-curricular out-of-class activities,” which further supports the sentiment that the engineering curriculum leaves little time for students to pursue co-curricular activities (Simmons et al., 2018). Interestingly, the study found that students significantly vary in their perceptions of what it means to be “highly active,” with significant variation in their reported hours.

The apparent lack of time for co-curricular participation may undermine the benefits of engagement, because students are not able to substantively explore co-curriculars to find the right fit or engage long enough to accumulate beneficial outcomes. For example, the NAS notes that while studies highlight retention and graduation rate benefits, they fail to address issues of initial motivation (National Academies of Sciences, 2017). It is reasonable to assume that finding the right co-curricular fit will require exploration of multiple co-curricular activities. Furthermore, it may require a period of engagement—weeks or months—before a student can meaningfully assess their interest and motivation for persisting. Thus, the issue of available time becomes a critical consideration to the design and facilitation of co-curricular programming.

#### Co-curricular motivation

The lack of time for co-curriculars relates to a third challenge—motivation. There is little evidence regarding the role of motivational factors in students’ decisions to participate in co-curriculars. Benefits that might be obtained from co-curricular activities are limited by the time available for participation. The ability of students to recognize specific achievement values and to assess tradeoffs of value and cost in different learning environments is central to this issue.

There is evidence in the literature that students are challenged to make such assessments. For example, Kirn and Benson (2018) reported that students who struggled to relate the value of certain assignments to their future tended to engage learning through those assignments superficially. They focused on completing assignments rather than learning from them (Kirn & Benson, 2018). The specific reasons for this difference remain an open question, but we hypothesize that students lack the ability to contextualize, assess, and internalize the benefits of co-curricular (and curricular) experiences in concrete terms. If students struggle with this in required course settings, it is reasonable that the unstructured and informal nature of co-curricular settings further exacerbates this issue. This research seeks to understand the role of motivational factors through the lens of Expectancy Value Theory.

### Expectancy-value theory as a conceptual framework

We used Expectancy-Value Theory (EVT) as a conceptual framework to investigate student motivation in co-curriculars (Atkinson, 1957; Eccles & Wigfield, 2002; Wigfield & Eccles, 2002; Wigfield et al., 2009). EVT relates several social, cultural, and affective factors to achievement-related choices of individual students, including their beliefs regarding the achievement value (intrinsic, attainment, and utility) and cost of participation in a particular task or activity (Wigfield et al., 2009).

EVT research has demonstrated that the motivational factors and interactions that drive student engagement and persistence in activities are complex (Brophy, 1999; Graham & Taylor, 2002; Wigfield & Eccles, 2002; Wigfield et al., 2009). For example, Jones et al. (2010) investigated the motivations of first year engineering students through the lens of EVT. They found that while both men and women have similar levels of value-related beliefs, both reported “enjoying engineering less and viewed it as less important and useful” by the end of the first year. Similar declines in motivational trajectories among engineering students in the first 2 years have also been reported by Robinson et al. (2019).

Furthermore, there are differences among genders and ethnicities pertaining to motivation constructs (Wigfield & Eccles, 2002). For instance, men and women may hold different expectancy beliefs for different activities (Eccles, 1987, 2007; Guo et al., 2015; Jones et al., 2010). Female students have been found to experience a low attainment (task completion) value as compared to their male peers in engineering education (Matusovich et al., 2010). The relative lack of women in engineering and other STEM majors has been attributed, in part, to these differences in beliefs and values and educators’ inability to act on those differences (Eccles, 2007; Guo et al., 2015).

Our lack of understanding of motivational differences among genders and ethnicities could be, in part, attributable to a lack of EVT research on the issue of cost, as reflected in required effort, opportunity costs, and emotional costs (Flake et al., 2015). Though under studied relative achievement values, more recent research underscores the importance of cost as a factor that impacts expectation and valuation (Flake et al., 2015; Perez et al., 2014; Robinson et al., 2019). Similarly, prior research has demonstrated that utility value becomes a more relevant consideration for students as they mature (Eccles & Wigfield, 1995), shifting student thinking away from motivations that are rooted in intrinsic or attainment value to considerations of what is valuable to future success.

Furthering our understanding of co-curricular motivations as it relates to educational value tradeoffs, such as utility vs. cost (e.g., time), is critical to designing and supporting co-curriculars and helping students to make better informed educational choices (Harackiewicz et al., 2014). This study examined how students perceive and why they engage or disengage in co-curriculars at a large public university. The study reported here investigates the co-curricular motivations among engineering undergraduates as reflected by EVT constructs.

## Methodology

To explore our research question introduced in "Study motivation: overview of preliminary study on student available time" section about student motivation for co-curriculars, we incorporated a sequential explanatory mixed methods design that consisted of a quantitative phase (survey) followed by a qualitative phase (individual interviews) (Creswell et al., 2003). This approach allowed us to further understand and explain statistical results by exploring participants’ views in more depth (Creswell et al., 2003; Tashakkori & Teddlie, 1998).

### Quantitative methods: survey instrument and analysis approach

#### Data collection

We developed a survey based on EVT survey items reported in the literature, adapted to the co-curricular context. The study involved engineering undergraduate students attending a large, public, research-intensive university in the northeast United States, where 19% are Women, 49.4% are White, 5.6% are Black or African American, 20.4% are Asian, 0.5% are Native Hawaii or other Pacific Islander, 6.7% are Hispanic/Latino, 9.3% are International, 2.5% are 2 or more ethnicities, and 5.5% are Unknown/Unreported. These percentages are based on data from the school’s office of undergraduate education for the semester in which this study took place and are demographically representative of engineering bachelor’s degrees awarded in 2018 according to ASEE (Roy, 2019). Three weeks prior to the end of the semester in Spring 2021, we distributed an online survey via the undergraduate engineering listserv. 110 students initiated the survey but only 74 participants completed it. Participants ranged in age from 18 to 31, with a mean age of 20.33.

#### Measures

Measures of students’ perceived achievement values and costs were based on EVT items adapted from multiple sources, including (Eccles & Wigfield, 1995; Flake et al., 2015) to best reflect the co-curricular context of the instrument. A total of 17 EVT related questions (5-point Likert scale) were included in the survey (two intrinsic value, three attainment value, two utility value, and 10 cost questions). The selection of items was based on their validity as reported in prior work. Revision to the wording of the items also considered work of (Battle & Wigfield, 2003) and (Perez et al., 2014). This study employed exploratory factor analysis (EVT) and scale construction methods on the 17 EVT related items. We used guidance from (Gliem & Gliem, 2003) regarding the strength of Cronbach’s Alpha as it relates to reliability of the measures. The original survey questions, our adaptations, the associated EVT constructs for each item, and Cronbach’s Alpha scores are provided in Appendix 1. The instrument on student perceived values have fair to good reliability as internal consistencies for constructs range from 0.47 to 0.8.

#### Data analysis

We used descriptive analyses and non-parametric tests to compare mean differences by co-curricular groups***.*** First, the descriptive analyses compare target variables with the main variables of interest (EVT constructs) to identify any potentially confounded relationships. The main analyses tested two hypotheses using a univariate approach: (1) there are significant mean differences in terms of perceived values and costs by student co-curricular participation status (college co-curricular participation only this year; not this year; both years; never); (2) there are significant mean differences in terms of perceived values and costs between students who participated and those who never participated (college co-curricular participation; never).

Second, we explored the EVT patterns by co-curricular participation status in college and students’ demographic groups (e.g., gender, transfer status, prior college co-curricular participation status, etc.). A non-parametric test was utilized to test associations between categorical (e.g., gender, transfer status, college co-curricular participation status, etc.) and numerical variables (e.g., index of perceived utility, index of perceived emotion cost, number of challenges, etc.). As the pilot data were non-normally distributed in a small sample, we selected the Mann–Whitney and the Kruskal–Wallis tests to compare mean differences by four co-curricular groups (college co-curricular participation only this year; not this year; both years; never) and by two groups (have participated, never participated) accordingly. Since the data were collected during the COVID-19 pandemic, the study aimed to determine whether results reveal variations in student engagement before and during the pandemic. Thus, it compared means of all seven EVT predictors for respondents who participated in co-curriculars in this and previous years, only in this year, in previous years but not this year, and never participated.

Similarly, the second Kruskal–Wallis test compared means of all seven EVT predictors by students’ academic standing groups (1 = first-year, 2 = sophomore, 3 = junior, 4 = senior). A set of Mann–Whitney tests was conducted to test the group mean differences of EVT indicators by students’ gender (1 = Male, 0 = Female), race (1 = White, 0 = non-White), pre-college co-curricular participation (1 = yes, 0 = no), and transfer status (1 = yes, 0 = no). As we performed multiple tests on a single set of data, we examined mean differences based on adjusted significance levels with Bonferroni correction to reduce type I errors.

### Qualitative data and data analysis

To further understand the motivation survey results, we conducted interviews via video conferencing tool. Interviewees were invited through the survey to voluntarily participate in the follow-up interview. Each interview was about 20 min in duration. The interviews were conducted by two authors of this study. The purpose of the interview was to allow respondents to speak openly and freely to uncover new insights (Creswell et al., 2003) and provide context for the survey results (see Appendix 2).

Each video-recorded interview was transcribed, and the data were initially coded using in vivo coding to capture participants’ lived experiences (Saldaña, 2014). Using Dedoose software, these initial codes were organized into categories and themes, using thematic analysis (Terry et al., 2017). To ensure the trustworthiness of the analysis, two coders (second author and a graduate student not affiliated with the study) reviewed, discussed, and finalized the coding schemes. The initial agreement between two coders was about 85%, and the discrepancies were resolved through a collaborative coding approach (Saldaña, 2014).

Of the 18 students who volunteered for the interview via survey response, only eight responded to an invitation and participated in the interview. Six interviewees had one (*n* = 2) or more than 2 years (*n* = 4) of co-curricular activities, while two interviewees had never participated in co-curriculars. Due to the small numbers of volunteers, we did not intentionally select interviewees.

## Results

### Quantitative findings

#### Descriptive analyses

Table 1 shows the descriptive statistics for the full sample, subsamples for respondents who participated in co-curricular activities, and those who never participated in co-curriculars. Under the Exploratory Factor Analysis process, the computed factor scores of motivation survey scales are standardized to a mean of 0 and the standard deviation of the distribution of factor scores is 1. Overall, there were more Male (68.9%) than Female respondents, and more White (76.4%) than non-White respondents. More than half of respondents reported participating in co-curricular activities before entering college (63.5%). In terms of students’ academic standing, 35.1% of respondents were first-year students, 12.2% were sophomores, 24.3% were juniors, and 28.4% were seniors. For students’ college co-curricular participation, 31.1% of respondents participated in co-curriculars only during the 2020–21 academic year; 5.4% participated in previous years but not this year; 40.5% participated in both this and previous years; and 23.0% never participated in co-curriculars.

**Table 1:** Descriptive statistics for co-curricular motivation survey

			By co-curricular group			
			Yes, has participated in co-curricular activities		No, never participated in co-curricular activities	
		*N*	*N*	%	*N*	%
**Demographics**						
Race	White (1)	55	46	83.64	9	16.36
	non-White (0)	17	9	52.64	8	47.06
Gender	Male (1)	51	39	76.47	12	23.53
	Female (0)	23	18	78.26	5	21.74
Transfer status	Yes (1)	9	5	55.56	4	44.44
	No (0)	65	52	80.00	13	20.00
Pre-college cocurricular	Yes (1)	47	38	80.85	9	19.15
	No (0)	27	19	70.37	8	29.63
Academic standing	First-year (1)	26	16	61.54	10	38.46
	Sophomore (2)	9	6	66.67	3	33.33
	Junior (3)	18	17	94.44	1	5.56
	Senior (4)	21	18	85.71	3	14.29
**Motivation Survey**	**N**	**Mean (S.D.)**	**N**	**Mean (S.D.)**	**N**	**Mean (S.D.)**
Intrinsic interest	74	0.00 (1.00)	57	− 0.04 (0.96)	17	0.14 (1.15)
Attainment	74	0.00 (1.00)	57	0.02 (1.04)	17	− 0.08 (0.89)
Utility	74	0.00 (1.00)	57	− 0.08 (1.01)	17	0.28 (0.94)
Task effort Cost	73	0.00 (1.00)	56	− 0.14 (0.93)	17	0.46 (1.11)
Outside effort Cost	74	0.00 (1.00)	57	− 0.20 (0.97)	17	0.67 (0.83)
Loss of alternatives	74	0.00 (1.00)	57	− 0.15 (0.98)	17	0.50 (0.91)
Emotion cost	74	0.00 (1.00)	57	− 0.07 (0.94)	17	0.22 (1.19)

In Table 1, the percentages of students who have participated in co-curricular activities (yes), and never participated in co-curricular activities are reported for dummy variables (race, gender, transfer status, pre-college co-curricular participation). Mean and standard deviation are reported for continuous variables

#### Motivational factors and their influence on student perceptions of co-curriculars

We sought to understand if students’ demographics (gender, race, transfer status, pre-college co-curricular participation, academic standing), and co-curricular participation status in college were correlated with their self-reported motivations (achievement values and costs) for co-curriculars. A series of non-parametric tests (the Mann–Whitney test for comparison between two groups; the Kruskal–Wallis test for comparison of more than two groups) were conducted. Epsilon square (*ε*^2^) was calculated based on the H-statistic as the measure of the Kruskal–Wallis test effect size (*ε*^2^ [*H*] = *H*/[(*n*^2^-1)/(*n* + 1)]), where H is the value obtained in the Kruskal–Wallis test; n is the total number of observations (Funder & Ozer, 2019; Tomczak & Tomczak, 2014). For the Mann–Whitney tests, *r*^2^ is calculated by z-score: (*r*^2^ = *z*^2^/*n*), where *z* is the *z* score obtained in the Mann–Whitney tests; *n* is the total number of observations. Only tests with significant results are reported (a summary of non-significant tests is provided as a Additional file 1).

Kruskal–Wallis tests of the four co-curricular participation statuses (college co-curricular participation (1) only this year, (2) not this year, (3) both years, (4) never) indicated that college curricular participation had a significant effect on students’ self-perceived outside effort cost, *H* (3) = 13.23, *p* = 0.004, *ε*^2^ = 0.18 (large effect size; see Table 2). There was a significant mean difference of students perceived outside effort cost among four co-curricular groups. Pairwise multiple comparisons with Bonferroni correction significance indicated that students who never participated in co-curriculars reported a statistically significant higher average score in the perceived cost of outside effort than those who participated in co-curriculars both this year and in previous years (*p* = 0.002).

**Table 2:** Kruskal–Wallis test: motivation pattern by four co-curricular groups

	NEVER participated	Participated in previous years NOT this year	Participated in ONLY this year (2020–2021)	Participated BOTH this and previous years		
	Mean (S.D.)	Mean (S.D.)	Mean (S.D.)	Mean (S.D.)	*H* value	ε^2^
Intrinsic interest	0.14 (1.15)	− 0.23 (1.03)	− 0.17 (0.98)	0.08 (0.95)	1.14	0.02
Attainment	− 0.08 (0.89)	− 0.55 (1.60)	− 0.06 (0.98)	0.17 (1.00)	1.36	0.02
Utility	0.28 (0.94)	− 0.51 (1.32)	− 0.01 (0.94)	− 0.08 (1.05)	2.22	0.03
Task effort cost	0.46 (1.11)	0.35 (1.00)	− 0.21 (0.97)	− 0.15 (0.91)	4.50	0.06
Task outside effort cost	0.67 (0.83)	0.22 (0.47)	0.04 (0.83)	− 0.43** (1.06)	13.23**	0.18
Loss of alternatives	0.50 (0.91)	− 0.29 (1.31)	0.04 (0.96)	− 0.27 (0.97)	5.98	0.08
Emotion cost	0.22 (1.19)	0.26 (1.14)	0.07 (0.87)	− 0.22 (0.97)	2.50	0.03

Pairwise comparisons are reported. The reference group is “students who never participated in co-curriculars

The significance values have been adjusted by Bonferroni correction for multiple tests; *p < 0.05, **p < 0.01

The non-parametric, Mann–Whitney test was performed to see whether there was a significant mean difference on motivation patterns between students who had co-curricular experiences at college and those who did not (Table 3). There was a significant effect on engineering students’ perceptions of two types of costs for co-curricular participation. Students who have participated in co-curriculars perceived less outside effort cost (z = -2.99, p < 0.01), and loss of valued alternatives (z = -2.27, p < 0.05) than those who never participated in co-curriculars. The effect sizes are small.

**Table 3:** Mann–Whitney U test: motivation Pattern by two co-curricular groups

	Participants who have participated in co-curricular activities		Participants who never participated in co-curricular activities		*z*-value	*r*^2^
	Mean	S.D	Mean	S.D		
Intrinsic interest	− 0.04	0.96	0.14	1.15	− 0.51	0.00
Attainment	0.02	1.04	− 0.08	0.89	− 0.50	0.00
Utility	− 0.08	1.01	0.28	0.94	− 1.24	0.02
Task effort cost	− 0.14	0.93	0.46	1.11	− 1.83	0.05
Task outside effort cost	− 0.20	0.97	0.67	0.83	− 2.99**	0.12
Loss of alternatives	− 0.15	0.98	0.50	0.91	− 2.27*	0.07
Emotion cost	− 0.07	0.94	0.22	1.19	− 0.67	0.01

*p < 0.05, **p < 0.01, ***p < 0.001; the analysis is based on the sample using listwise deletion

Thesequantitative results show that there is a significant difference in perceptions of cost of co-curricular participation among those who have and have not participated. More specifically, the results suggest that students who have never participated believe that participation is not possible because of current commitments (task outside effort cost) and concern about other interests (loss of alternatives). Notably, the results suggest that there is no statistical difference between groups regarding beliefs about the effort necessary for co-curricular activities themselves. Similarly, there is no statistical difference in perceptions about achievement values (intrinsic, attainment, utility) between students who have and have not participated.

### Qualitative findings: themes from participant interviews

From the follow-up interviews, we were able to further understand the quantitative findings. Ninety initial codes emerged through in vivo coding and were merged into nineteen final codes as shown in Appendix 3 with representative quotes. The nineteen codes were then categorized into five themes as shown in the Table 4: (1) value of co-curriculars, (2) challenges in co-curriculars, (3) challenges in engineering classes, (4) co-curricular challenges during the pandemic, and (5) general perceptions of co-curriculars. We use pseudonyms when referring to specific interviewees.

**Table 4:** Categories and themes from interview data analysis

Themes	Codes
Value of co-curriculars	Being able to connect with coursework
	Connecting with partners/senior professionals/teamwork
	Receiving learning support from peers
	Learning leadership
	Opening up future opportunities/job seeking
	Opportunities to reflect on personal interests in engineering majors
	Authentic engineering experiences
Challenges in engineering courses	Difficult subjects/courses to follow
	Disconnect between coursework and practice
	Lack of prior knowledge in the course/subject
Challenges in co-curriculars	Time related challenges
	Challenging group work
Co-curricular challenges during the pandemic	Organizational challenges
	Lack of working space during the pandemic
	Economic/funding deficit/job challenges
	Not being able to do in-person collaboration
General perceptions of co-curriculars	Highly enjoyable/beyond expectation/priority
	Meeting expectation/interesting as expected
	Require autonomous engagement

The operational definition for each theme is as follows: *Value of co-curriculars* reflects interviewees’ talk about the perceived benefits of participating in co-curriculars; *Challenges in engineering courses* reflects interviewees’ talk related to difficulties encountered in engineering courses; *Challenges in co-curriculars* reflects interviewees’ talk related to difficulties encountered while participating in co-curriculars; *Co-curricular challenges during the pandemic* reflects interviewees’ challenges in participating co-curriculars particularly during the pandemic; *General perceptions of co-curriculars* reflects interviewees’ talk about their perceptions on co-curriculars whether they had participated or not in co-curriculars.

#### Theme: perceived values of co-curriculars

Regarding the perceived value of co-curriculars, students with and without co-curriculars recognized value but shared different perspectives. The interviewees who had not participated in co-curriculars believed participating in co-curriculars would open opportunities for them to get internships or secure a job in the future. However, they could not provide more specific insights as to why co-curriculars would be beneficial to their employability.

Those students who had co-curricular experiences, on the other hand, shared a variety of perceived values that fit into seven themes (Table 4). They valued opportunities to connect engineering coursework with authentic and hands-on learning experiences. For example, Monica, who had more than 2 years of experience in co-curriculars enjoyed participating, because she was able to “*apply theories I learned from the coursework into the co-curriculars project*” by doing a “*hands-on*” project. She said, *“I can have practical experience and that’s outside of just doing theoretical stuff in the classroom.”*

In addition, they described co-curriculars as a place to build meaningful networks by collaborating with others. The value of these networks extended beyond co-curricular work to include peer support on engineering coursework. Three interviewees shared that being in co-curricular activity groups provided them with opportunities to develop social and leadership skills through networking, which they expected to be helpful for their future job seeking. Aron, an aerospace engineering major, found co-curriculars very beneficial for his job seeking in the future, because the club provided him with multiple opportunities to connect with senior members of the club as well as industry leaders: “*Working with a team of people who were a lot more experienced than you, gives you guidance when you're working on this kind of thing. We’re our own support group and senior members look out for younger members.*”

Finally, some interviewees described aspects of co-curriculars that map to practice. This included opportunities to pursue personal interests in their majors and be part of an authentic experience. One interviewee described a real-world style workflow and accountability “*where people that are available to do a task, make a commitment to that timeframe and they do what they're expected to do or seek help if they're unable to accomplish.”*

#### Theme: challenges of the curriculum

Both students with and without co-curricular experience shared common challenges of engineering courses. They perceived engineering courses as difficult and time-consuming and acknowledged a heavy coursework load. They also shared that they were challenged when they could not connect the course content to the real-world and when they felt a lack of prior knowledge in the subject area or relevant mathematics. For the two students who had not participated in co-curriculars, the main reason that they have not been able to participate is because they “*did not have time”* given that in their engineering courses it is “r*eally hard to absorb the concepts and the course load is really tough.*” One interviewee wished “*there was a lot more time.*” This qualitative finding aligns with the quantitative finding that outside effort cost (i.e., course load) is too high and prevents students who have not participated from engaging co-curriculars.

#### Theme: challenges in co-curriculars—general and pandemic related

Co-curriculars have their challenges too. Students described difficulty finding common times to meet and time to complete individual co-curricular tasks. This challenge was related to the time necessary for completing coursework.

Such challenges were more acute during the COVID-19 pandemic, because in-person meetings and access to existing spaces and resources (e.g., meeting rooms, machine shop) were restricted. In addition, the pandemic led to budget cuts and funding deficits. As described by one interviewee, “*the biggest challenge with COVID we basically had was that there were a lot more restrictions, so we only had- could have five people in our lab space at any one time.”* Another interviewee said that they had* “a rotation of individuals going through at all different timeframes due to limited lab capacities.”*

#### Theme: general perceptions of co-curricular participants

Despite such challenges, students with co-curricular experience generally reported that it was worth those difficulties. Compared to taking courses to get grades, they enjoyed greater autonomy and the opportunity to show initiative that co-curriculars allow. One student described their experiences as *“amazing,”* and they were willing to put higher priority on co-curriculars than some of the curricular requirements of their major.

## Discussion

The quantitative and qualitative findings reported in "Results" section surface insights regarding student perceptions of co-curriculars as it relates to motivational factors of *cost* and *value* of participation. We discuss each of these individually, considering nuanced aspects of these factors that reveal potential misalignment between student perceptions and reality. We conclude with implications that might help to mitigate such misalignment and opportunities for additional research necessary to improve our understanding and student access to co-curricular opportunities.

### Perceived ‘high-cost’ for co-curricular engagement

Prior research has suggested that the rigors of the engineering curriculum may lead many students to conclude that they do not have time to participate in co-curriculars (Lichtenstein et al., 2010; Miller et al., 2018; Simmons et al., 2017, 2018). Indeed, the co-curricular motivation survey in this study found that students who have never participated in co-curriculars are more likely to perceive a higher cost as it relates to outside effort and loss of valued alternatives than their peers who have previously participated. That is, these students believe that participation will take away too much time from current obligations and opportunities. This finding was backed by student interviews in which they described those perceptions and the time necessary for coursework as a barrier to co-curricular participation.

This quantitative and qualitative finding suggests that, regardless of the actual time they may have available, beliefs about the high cost of participation will deter many students from ever engaging co-curriculars in the first place. Yet, because they have not been involved, they cannot be sure how much time is required. Regarding the question on the time necessary for co-curricular participation, the six interviewees with co-curricular experiences reported the time ranged from four to 20 h per week, and the median was 10 h. The two interviewees without co-curricular experiences expected they would need 10 and 30 h per week, respectively.

Perceptions of “high cost” as a deterrent is a significant issue because if co-curricular experiences are valuable in terms of technical and professional skill development, and in contributing to formation of engineering identity and sense of belonging (Andrews et al., 2021), the lack of engagement is a missed opportunity for many students. Furthermore, the high cost reinforces existing barriers that may disproportionately impact marginalized groups, which engineering programs have traditionally struggled to attract and retain.

### The value of co-curriculars—professional development vs. social networks

The lack of statistically significant differences on achievement value factors (intrinsic, attainment, and utility value) among students who have and have not participated is also potentially important. This finding invites two possible interpretations. First, it suggests that both students who have and have not participated in co-curriculars generally have the same perceptions of co-curricular value. This is important in so much as it provides potential leverage for encouraging initial and persistent engagement if those values are institutionally recognized and reinforced.

A second, more nuanced interpretation is that even after students initially engage in co-curriculars, they do not perceive value in terms future utility. As shown in Table 3, students who have participated in co-curriculars reported a lower utility value (− 0.08) than those who have not (0.28). Students who participated in previous years but not this year have an even lower utility rating (− 0.51, Table 2). While not statistically significant, it stands out as counter to our expectation. We reasoned that students who participated would recognize such value at a rate higher than their non-participating peers. This is not incongruent with findings of other researchers who explored motivation among engineering and STEM students more generally (Flake et al., 2015; Jones et al., 2010; Kirn & Benson, 2018; Perez et al., 2014; Robinson et al., 2019). Educational experiences, both in and out of the classroom, often do not do enough to help students contextualize their experiences, such that they can recognize specific forms of learning and its value in terms of the profession. The interview data support this interpretation. Some of the interviewees mentioned co-curriculars as helpful to applying theories they learned from engineering courses but did not describe how that application might extend to practice. Nor did they describe development of specific technical and professional competencies like those found in the literature (Carter et al., 2016; Shehata, 2015; Young et al., 2014). While studies have described specific competency development recognized by students through co-curriculars (e.g., Garrett et al., 2021), it is interesting that in our interviews, students were not more explicit about specific competencies.

From our study results and interviews, we learned students’ assessment of the value of such experiences had more to do with having something to show on their resume. As students get closer to job seeking points of the curriculum (e.g., internships, co-ops, entry-level positions), the perceived value of co-curriculars may be more about signaling “experience” to employers or in networking and making connections with individuals already established in industry. This justification for persisting in co-curriculars is also described by (Stevens et al., 2008) as a reason among students at an urban private university who “believed that participation in certain clubs would help in networking and thereby future employment in the profession following college.” Among the students that we interviewed, this reason was explicitly cited by two students.

Another reason for engagement in co-curriculars we observed from the current study was related to socializing and forming friendships. These social connections can be an important element of how students “navigate” the engineering curriculum (Stevens et al., 2008). It provides a community of like-minded students who are having similar experiences, providing an emotional and psychological support group among individuals who are “in this together.” Among the students we interviewed, the sense of community and social networking with peers also emerged as an important factor in persistent participation. While we agree that sense of community that co-curriculars can provide is valuable, we see it as equally important for students to recognize and articulate other forms of value from co-curriculars.

### Conclusions: Implications for the design and support of co-curriculars

Our findings suggest that engineering students’ access to and motivation for co-curricular engagement may not be aligned with the intentions and aspirations of the institutions and professionals that encourage them. Where departments, schools, and alumni encourage co-curricular involvement to extend student learning in ways that relate to the profession and enjoy a more holistic educational experience, student perceptions suggest a critical gap. We consider implications for bridging this gap as it relates to co-curricular value recognition and improving access.

#### Implications for improving co-curricular access (overcoming high cost)

Co-curriculars are something that students often pursue voluntarily, in addition to their already busy schedules. Thus, the high cost of getting involved (real or perceived) limits access and represents an inequity that educational institutions ideally seek to mitigate. This misalignment highlights a need to (1) increase understanding of the factors that affect engineering student engagement and learning in co-curriculars, and (2) operationalize that increased understanding to inform student decision making and institutional policy as it relates to greater integration of co-curriculars for all students.

At the level of individual student, we see a need for greater evidence about students’ use of time. While there is significant research related to complementary issues, such as self-regulation (English & Kitsantas, 2013; Galand et al., 2010), a search for literature related to student time allocation on Google scholar over the past decade yielded few results with a time study element (i.e., keeping a time journal (Ayers et al., 2012)). None were specific to engineering students. Our prior study (Olewnik & Sreeram, 2021) suggests that there is time (in aggregate) for many students to pursue co-curriculars, but it does little to help us understand how available time is distributed or how students make judgements and tradeoffs in dividing their time between curricular and co-curricular learning opportunities. Research that tracks the ways in which students allocate their time throughout the week (and potentially the related stress) would be valuable in developing a more precise understanding of time use and its impact on the student experience. Such information would inform the development of interventions that support students in making tradeoffs to balance their formal and informal educational experiences.

Beyond the individual student, we need to consider the design of the co-curricular environments and support staff. Because there is limited research on co-curricular environments and activities (Simmons et al., 2018) it is difficult to inform the design and support of those environments to overcome engagement barriers for students, which are linked to time and motivation factors. For example, understanding the time available overall and the duration of openings in student schedules might inform the design of individual co-curricular learning activities, like learning how to use equipment in a university makerspace. However, other co-curricular activities, like technical competition projects, require collaboration among students and significant student scheduling conflicts during the day may make meeting infeasible. This leads to students meeting after normal class hours, but this may mean that support facilities (e.g., machine shops and makerspaces) are not available, because they close when the staff leave. This may undermine student motivation and make them feel unsupported.

The results of this study should motivate a discussion among a variety of stakeholders, including students, faculty, academic advisors, departmental curriculum planners, school administrators, and co-curricular programs. As described by Lee and Matusovich (2016), institutional support can include a range of services, programs, and activities that impact the undergraduate experience inside and outside of the classroom. Adoption of their framework to guide research, in combination with research on student motivation and time studies, would help in developing a more robust understanding of the undergraduate student experience, including the role of co-curriculars. This could lead to interventions and curricular design that supports opportunities for more students to explore engineering and individualize their learning through experiences that are traditionally reserved for co-curricular settings, while still meeting graduation requirements and accreditation criteria, like ABET. We know this is already possible, as many institutions allow students to earn credit for undergraduate research and student club projects through existing mechanisms like independent study and capstone design. We believe that allowing more co-curricular experiences to fulfill academic requirements deserves greater investigation. This would improve access to valuable learning experiences for all without putting additional demands on students’ already busy academic schedules. A tighter integration and systematic discussion among institutional stakeholders, informed by the intricacies of individual student time constraints and motivation, is necessary to provide these experiences at scale, and overcome inequities in the student experience.

#### Implications for supporting co-curricular value recognition

This study suggests that students struggle to attribute specific value to co-curriculars. Therefore, while there is evidence from prior studies that valuable learning takes place in co-curricular settings, students may not recognize those outcomes nor their relevance to the profession. This may undermine consistent engagement in co-curriculars. To overcome this potential for disengagement, developing interventions that guide students in recognizing the most likely learning outcomes from different co-curriculars is needed to help them in making decisions about which co-curriculars to pursue.

In addition, once students are engaged in co-curriculars, there is a need to support reflection on those experiences, such that students can draw on important lessons later, like in interview settings (Olewnik et al., 2021). Such reflection could be naturally integrated as part of the portfolio of experiences that students should be building throughout their education.

### Limitations

There are a few limitations of our study, which are not unexpected for a pilot study. First, we have a relatively homogeneous sample (the majority are white students). For data collection and improvement, future studies should consider and collect a larger sample from students with diverse demographic backgrounds (and from multiple institutions). In addition, some factors and profiles are not captured by the pilot data that should be considered in the future study (e.g., socioeconomic status, school profiles, students-level outcomes). Deeper dive data collection with students is required to understand time management skills, and other aspects of motivation in a qualitative sense (better characterization of the survey factors). Furthermore, future studies should develop and use more reliable survey instruments. The instruments on student perceived values have fair to good reliability. In the current study, we focus on descriptive analysis of motivation patterns. Future studies may examine whether and how time and motivation in co-curricular activities may promote student learning outcomes by trend analysis (e.g., regression analysis, growth modeling).

### Supplementary Information

**Additional file 1. **SPSS output: non-significant results for group comparisons.

## Data Availability

Data used for the time study summarized in "Study motivation: overview of preliminary study on student available time" section are property of the institution and are not publicly available. Data resulting from the survey in "Qualitative data and data analysis" section will be archived and publicly available through a library repository at the institution of the corresponding author. The corresponding author should be contacted to inquire about accessing the underlying data.

## Appendix

### Appendix 2. Interview Protocol

To Students with Co-curricular activities:

1. Student Background Experiences

- What made you decide to major in engineering/STEM? Has your experience met your expectations?

2. Student Motivation and Satisfaction

- What co-curricular did you participate in this year?
- Why do you participate in co-curriculars?
- What did you expect and did your experience meet expectations?
- Where do you see co-curriculars fitting into your future career/school?

3. Student Experiences

- What types of challenges do you face in co-curriculars?
- How much time do you spend on co-curriculars each week? Would you want to spend more or less time? Has co-curricular involvement taken away time from other activities (class, studying, hobbies, working, etc.)?

To Students without Co-curricular activities:

1. Student Background Experiences

- What made you decide to major in engineering/STEM? Has your experience met your expectations?

2. Student Motivation and Satisfaction

- Have you ever considered participating in co-curriculars? What keeps you from engaging?
- How do you think about the benefits of co-curriculars for your future career or academics?

3. Student Experiences

- How much time do you think co-curriculars would require? Do you think this would take too much time from other responsibilities?

## Abbreviations

ANOVA: Analysis of variance
ASCE: American Society of Civil Engineers
ATUS: American Time Use Study
BLS: Bureau of Labor and Statistics
BMES: Biomedical Engineering Society
EVT: Expectancy-Value Theory
HSD: Honest Significant Difference
Hwk: Time spent preparing for class
NAE: National Academy of Engineering
NAS: National Academies of Sciences
NSSE: National Survey of Student Engagement
REU: Research Experiences for Undergraduates
RQ: Research Question
RT: Residual time
SAE: Society of Automotive Engineers
SEAS: School of Engineering and Applied Sciences
Slp: Time allocated to sleep
Soc: Time spent socializing and relaxing
STEM: Science, Technology, Engineering, and Mathematics
Trv: Weekly commute time for students
UR: Undergraduate Research
Wrk: Time spent working
