# Supplementary material for: Co-curricular engagement among engineering undergrads: do they have the time and motivation?
Source: Int J STEM Educ. 2023 Apr 5;10(1):27. doi: 10.1186/s40594-023-00410-1 (PMC10074349; doi:10.1186/s40594-023-00410-1)
Supplement: Supplementary file 1 — Additional file 1. SPSS output: non-significant results for group comparisons. [file 40594_2023_410_MOESM1_ESM.docx]

**Additional file 1**

**SPSS output: non-significant results for group comparisons**

**Nonparametric Tests for gender (Male vs. Female)**

|  | | | | | |
| --- | --- | --- | --- | --- | --- |
|  | Null Hypothesis | | Test | Sig.^a,b^ | Decision |
| 1 | The distribution of factor score: intrinsic interest is the same across categories of gender. | | Independent-Samples Mann-Whitney U Test | .838 | Retain the null hypothesis. |
| 2 | The distribution of factor score: attainment is the same across categories of gender. | | Independent-Samples Mann-Whitney U Test | .783 | Retain the null hypothesis. |
| 3 | The distribution of factor score: utility is the same across categories of gender. | | Independent-Samples Mann-Whitney U Test | .112 | Retain the null hypothesis. |
| 4 | The distribution of factor score: task effort cost is the same across categories of gender. | | Independent-Samples Mann-Whitney U Test | .838 | Retain the null hypothesis. |
| 5 | The distribution of factor score: task outside effort cost is the same across categories of gender. | | Independent-Samples Mann-Whitney U Test | .307 | Retain the null hypothesis. |
| 6 | The distribution of factor score: loss of alternatives is the same across categories of gender. | | Independent-Samples Mann-Whitney U Test | .869 | Retain the null hypothesis. |
| 7 | The distribution of factor score: emotion cost is the same across categories of gender. | | Independent-Samples Mann-Whitney U Test | .691 | Retain the null hypothesis. |
|  | | a. The significance level is .050. | | | |
|  | | b. Asymptotic significance is displayed. | | | |

**Nonparametric Tests for race (White v.s. Non-white)**

|  | | | | | |  | | |
| --- | --- | --- | --- | --- | --- | --- | --- | --- |
|  | Null Hypothesis | | Test | Sig.^a,b^ | Decision | | |  |
| 1 | The distribution of factor score: intrinsic interest is the same across categories of race: White v.s.Non-white. | | Independent-Samples Mann-Whitney U Test | .796 | Retain the null hypothesis. | | |  |
| 2 | The distribution of factor score: attainment is the same across categories of race: White v.s.Non-white. | | Independent-Samples Mann-Whitney U Test | .253 | Retain the null hypothesis. | | |  |
| 3 | The distribution of factor score: utility is the same across categories of race: White v.s.Non-white. | | Independent-Samples Mann-Whitney U Test | .205 | Retain the null hypothesis. | | |  |
| 4 | The distribution of factor score: task effort cost is the same across categories of race: White v.s.Non-white. | | Independent-Samples Mann-Whitney U Test | .256 | Retain the null hypothesis. | | |  |
| 5 | The distribution of factor score: task outside effort cost is the same across categories of race: White v.s.Non-white. | | Independent-Samples Mann-Whitney U Test | .263 | Retain the null hypothesis. | | |  |
| 6 | The distribution of factor score: loss of alternatives is the same across categories of race: White v.s.Non-white. | | Independent-Samples Mann-Whitney U Test | .369 | Retain the null hypothesis. | | |  |
| 7 | The distribution of factor score: emotion cost is the same across categories of race: White v.s.Non-white. | | Independent-Samples Mann-Whitney U Test | .498 | Retain the null hypothesis. | | |  |
|  | | a. The significance level is .050. | | | | |  |  |
|  | | b. Asymptotic significance is displayed. | | | | | | |

**Nonparametric Tests for transfer status (Yes vs. No)**

|  | | | |  |  |
| --- | --- | --- | --- | --- | --- |
|  | Null Hypothesis | Test | Sig.^a,b^ | Decision |  |
| 1 | The distribution of factor score: intrinsic interest is the same across categories of transfer status. | Independent-Samples Mann-Whitney U Test | .946 | Retain the null hypothesis. |  |
| 2 | The distribution of factor score: attainment is the same across categories of transfer status. | Independent-Samples Mann-Whitney U Test | .540 | Retain the null hypothesis. |  |
| 3 | The distribution of factor score: utility is the same across categories of transfer status. | Independent-Samples Mann-Whitney U Test | .954 | Retain the null hypothesis. |  |
| 4 | The distribution of factor score: task effort cost is the same across categories of transfer status. | Independent-Samples Mann-Whitney U Test | .539 | Retain the null hypothesis. |  |
| 5 | The distribution of factor score: task outside effort is the same across categories of transfer status. | Independent-Samples Mann-Whitney U Test | .079 | Retain the null hypothesis. |  |
| 6 | The distribution of factor score: loss of alternatives is the same across categories of transfer status. | Independent-Samples Mann-Whitney U Test | .226 | Retain the null hypothesis. |  |
| 7 | The distribution of factor score: emotion cost is the same across categories of transfer status. | Independent-Samples Mann-Whitney U Test | .249 | Retain the null hypothesis. |  |
| a. The significance level is .050. | | | | | |
| b. Asymptotic significance is displayed. | | | | | |

**Nonparametric Tests for pre-cocurricular participation status (Yes v.s. No)**

|  | | | |  |  |
| --- | --- | --- | --- | --- | --- |
|  | Null Hypothesis | Test | Sig.^a,b^ | Decision |  |
| 1 | The distribution of factor score: intrinsic interest is the same across categories of pre-college co-curricular experience. | Independent-Samples Mann-Whitney U Test | .124 | Retain the null hypothesis. |  |
| 2 | The distribution of factor score: attainment is the same across categories of pre-college co-curricular experience. | Independent-Samples Mann-Whitney U Test | .761 | Retain the null hypothesis. |  |
| 3 | The distribution of factor score: utility is the same across categories of pre-college co-curricular experience. | Independent-Samples Mann-Whitney U Test | .888 | Retain the null hypothesis. |  |
| 4 | The distribution of factor score: task effort cost is the same across categories of pre-college co-curricular experience. | Independent-Samples Mann-Whitney U Test | .481 | Retain the null hypothesis. |  |
| 5 | The distribution of factor score: task outside effort cost is the same across categories of pre-college co-curricular experience. | Independent-Samples Mann-Whitney U Test | .565 | Retain the null hypothesis. |  |
| 6 | The distribution of factor score: loss of alternatives is the same across categories of pre-college co-curricular experience. | Independent-Samples Mann-Whitney U Test | .395 | Retain the null hypothesis. |  |
| 7 | The distribution of factor score: emotion cost is the same across categories of pre-college co-curricular experience. | Independent-Samples Mann-Whitney U Test | .835 | Retain the null hypothesis. |  |
| a. The significance level is .050. | | | | | |
| b. Asymptotic significance is displayed. | | | | | |

**Nonparametric Tests for academic standing groups (freshman/sophomore/junior/senior)**

|  | | | | |
| --- | --- | --- | --- | --- |
|  | Null Hypothesis | Test | Sig.^b,c^ | Decision |
| 1 | The distribution of factor score: intrinsic interest is the same across categories of aca_stand. | Independent-Samples Mann-Whitney U Test | .^a^ | Unable to compute. |
| 2 | The distribution of factor score: intrinsic interest is the same across categories of aca_stand. | Independent-Samples Kruskal-Wallis Test | .233 | Retain the null hypothesis. |
| 3 | The distribution of factor score: attainment is the same across categories of aca_stand. | Independent-Samples Mann-Whitney U Test | .^a^ | Unable to compute. |
| 4 | The distribution of factor score: attainment is the same across categories of aca_stand. | Independent-Samples Kruskal-Wallis Test | .647 | Retain the null hypothesis. |
| 5 | The distribution of factor score: utility is the same across categories of aca_stand. | Independent-Samples Mann-Whitney U Test | .^a^ | Unable to compute. |
| 6 | The distribution of factor score: utility is the same across categories of aca_stand. | Independent-Samples Kruskal-Wallis Test | .049 | Reject the null hypothesis.  (Here shows the sig results without Bonferroni correction. However, with the correction, it becomes non-significant.) |
| 7 | The distribution of factor score: task effort cost is the same across categories of aca_stand. | Independent-Samples Mann-Whitney U Test | .^a^ | Unable to compute. |
| 8 | The distribution of factor score: task effort cost is the same across categories of aca_stand. | Independent-Samples Kruskal-Wallis Test | .726 | Retain the null hypothesis. |
| 9 | The distribution of factor score: task outside effort cost is the same across categories of aca_stand. | Independent-Samples Mann-Whitney U Test | .^a^ | Unable to compute. |
| 10 | The distribution of factor score: task outside effort cost is the same across categories of aca_stand. | Independent-Samples Kruskal-Wallis Test | .549 | Retain the null hypothesis. |
| 11 | The distribution of factor score: loss of alternatives is the same across categories of aca_stand. | Independent-Samples Mann-Whitney U Test | .^a^ | Unable to compute. |
| 12 | The distribution of factor score: loss of alternatives is the same across categories of aca_stand. | Independent-Samples Kruskal-Wallis Test | .684 | Retain the null hypothesis. |
| 13 | The distribution of factor score: emotion cost is the same across categories of aca_stand. | Independent-Samples Mann-Whitney U Test | .^a^ | Unable to compute. |
| 14 | The distribution of factor score: emotion cost is the same across categories of aca_stand. | Independent-Samples Kruskal-Wallis Test | .977 | Retain the null hypothesis. |

a. The group field does not have exactly two values.

b. The significance level is .050.

c. Asymptotic significance is displayed.
